# A tale of two species: the origin of the Cantonese cancer in southern China

**DOI:** 10.1093/nsr/nwae470

**Published:** 2025-01-27

**Authors:** Rasmus Nielsen

**Affiliations:** Department of Integrative Biology and Department of Statistics, University of California, Berkeley, USA; GeoGenetics Centre, Globe Institute, University of Copenhagen, Denmark

**Keywords:** species, Cantonese, Cancer, Epstein-Barr virus

Epstein-Barr virus (EBV), the first human oncogenic virus discovered in 1964, initially drew attention for its role in Burkitt lymphoma [1]. Today it is recognized as the causative agent of infectious mononucleosis and is linked to several malignancies of epithelial and lymphocytic origin [2]. EBV-related diseases exhibit notable geographic patterns, leaving scientists puzzled about the virus's evolution in humans and the geographic differences in disease manifestation. In particular, the EBV-associated disease nasopharyngeal carcinoma (NPC) is endemic to southern China, earning it the nickname ‘Cantonese cancer’ [3]. The incidence rate of NPC in southern China is almost 20-fold higher than the rest of the world due to the presence of a highly pathogenic EBV strain (known as the high-risk strain) strongly linked to NPC [4]. Surprisingly, researchers discovered a clonal substrain within the high-risk strain which has rapidly expanded across southern China. Until now, the origin of this clonal strain and the factors driving its proliferation have remained enigmatic.

A new study in *National Science Review* by Zhang *et al.* [5] provides new insights into the diversification of EBV and the origins of the clonal strain. Zhang *et al.* [5] discovered that EBV's evolutionary history closely mirrors that of humans migrating out of Africa. Such an evolutionary pattern, in which the genetic diversification of the pathogen largely follows that of the host, has previously been observed in *Helicobacter pylori* and *Mycobacterium tuberculosis* [4,6,7], but is surprising to find in a virus. Most viruses tend to evolve fast and with substantial horizontal transmission and it is, therefore, unlikely that their evolutionary histories would mimic that of their hosts. The fast spread of the virus should rapidly erase the geographic pattern generated by the migration history of the host. However, herpes viruses such as EBV tend to evolve slowly. EBV may also have relatively more vertical transmissions than many other viruses because of the strong latency of the infection. Whatever the cause, Zhang *et al.* [5] demonstrate that EBV and humans have diversified together since the human out-of-Africa migration, and perhaps much earlier than that.

Zhang *et al.* [5] also show that the clonal strain from southern China has a mosaic genomic structure, formed through recombination between northern and southern East Asian EBV strains. Using molecular dating, they date the recombination event to ∼4000 years ago. This date coincides with historical human migration patterns in East Asia. Between 3000 and 5000 years ago northern Chinese populations began migrating southward, admixing with southern groups [8]. This admixture likely facilitated the EBV strain recombination that gave rise to the clonal strain. The result is a cancer endemic in East Asia, with an unusual geographic pattern explained by the evolutionary origins of the causative strain.

The analyses by Zhang *et al.* [5] show that strong Darwinian selection has driven the clonal strain's expansion, with an estimated fitness advantage of 4.2%. The spread of this strain over the past 2000 years predicts more than a doubling in the NPC incidence rate from 0.069% to the current 0.163% in southern China due to this strain alone. Recombination between different viral strains is a well-known driver of new virulent strains in viruses such as *Influenza* (in the form of reassortment). Due to the study of Zhang *et al.* [5] we now know that the high NPC incidence rate in southern China, at least in part, is due to a similar emergence of a new strain of EBV virus through recombination 4000 years ago. What we do not know is why this is happening particularly in southern China, but the results of Zhang *et al.* [5] would suggest that it may not be a consequence of specific environmental or genetic factors. Rather, it is a historical contingency—a product of the randomness of the processes governing viral evolution. Similar geographic variation in other slowly evolving oncogenic viruses may help explain regional differences in the incidence rates of other types of cancer.
